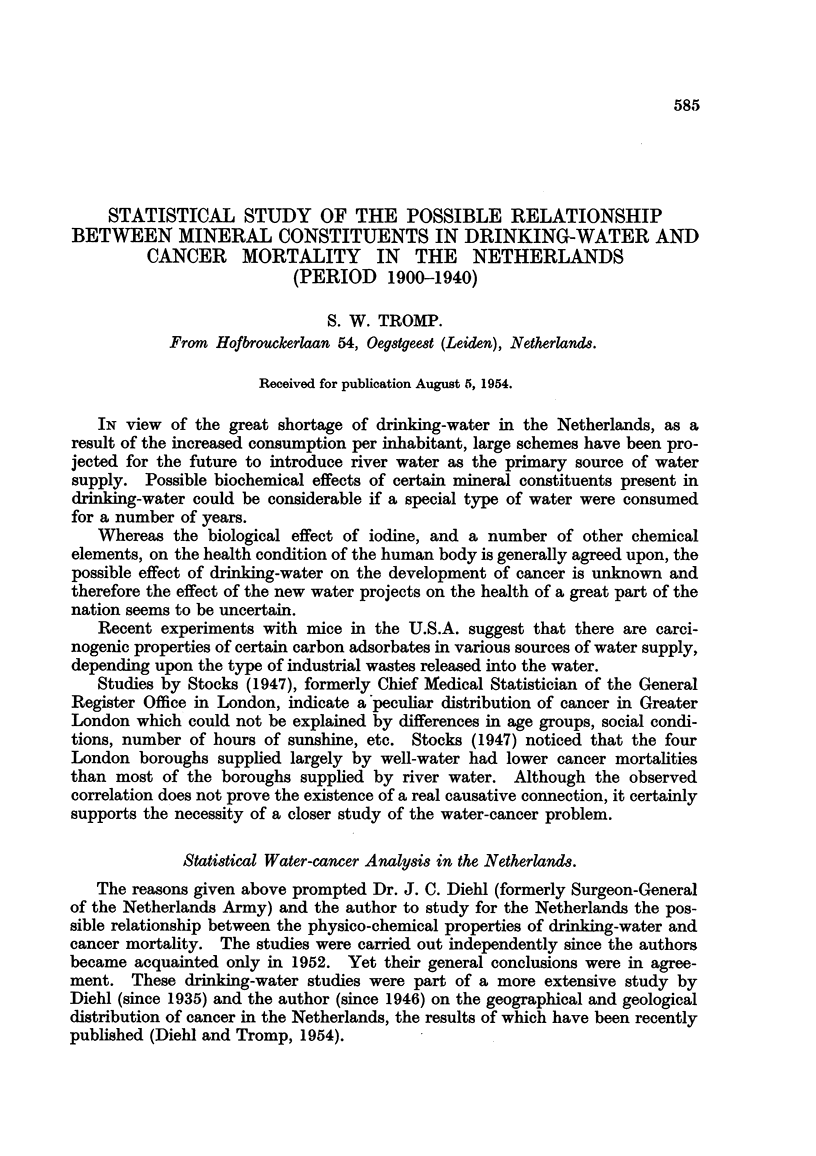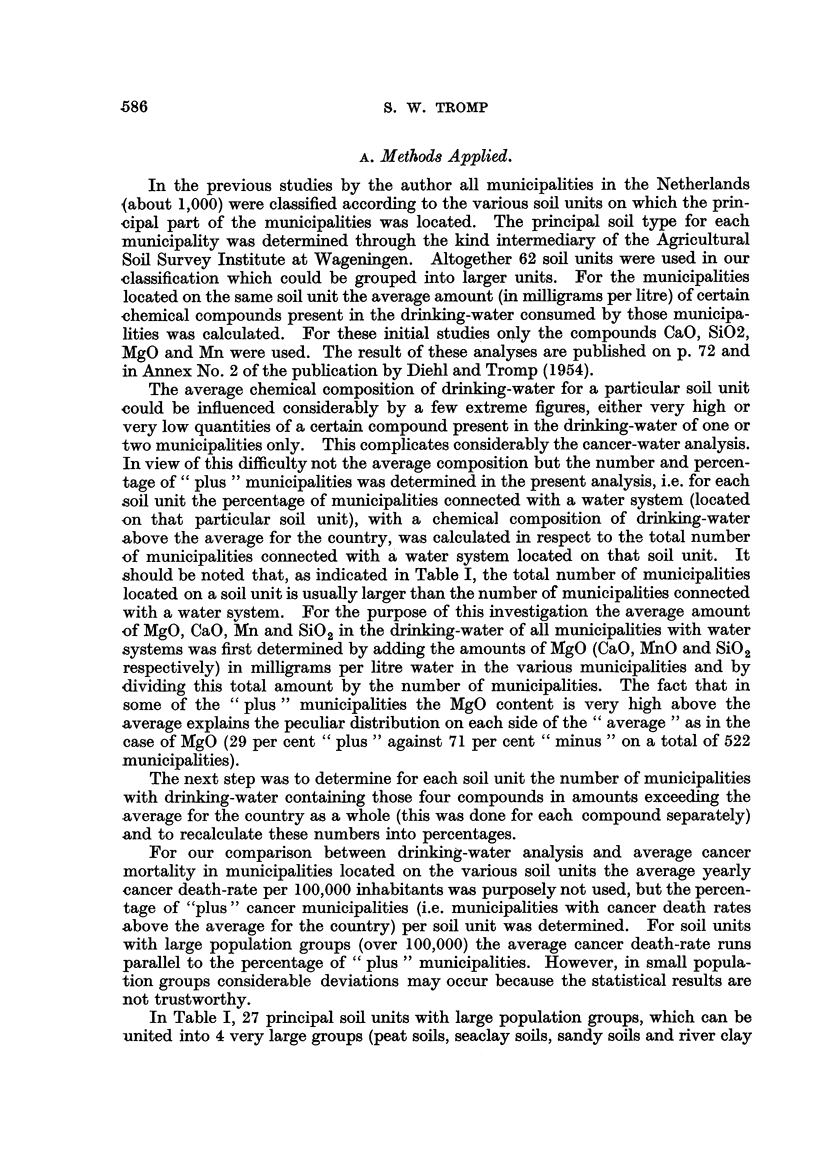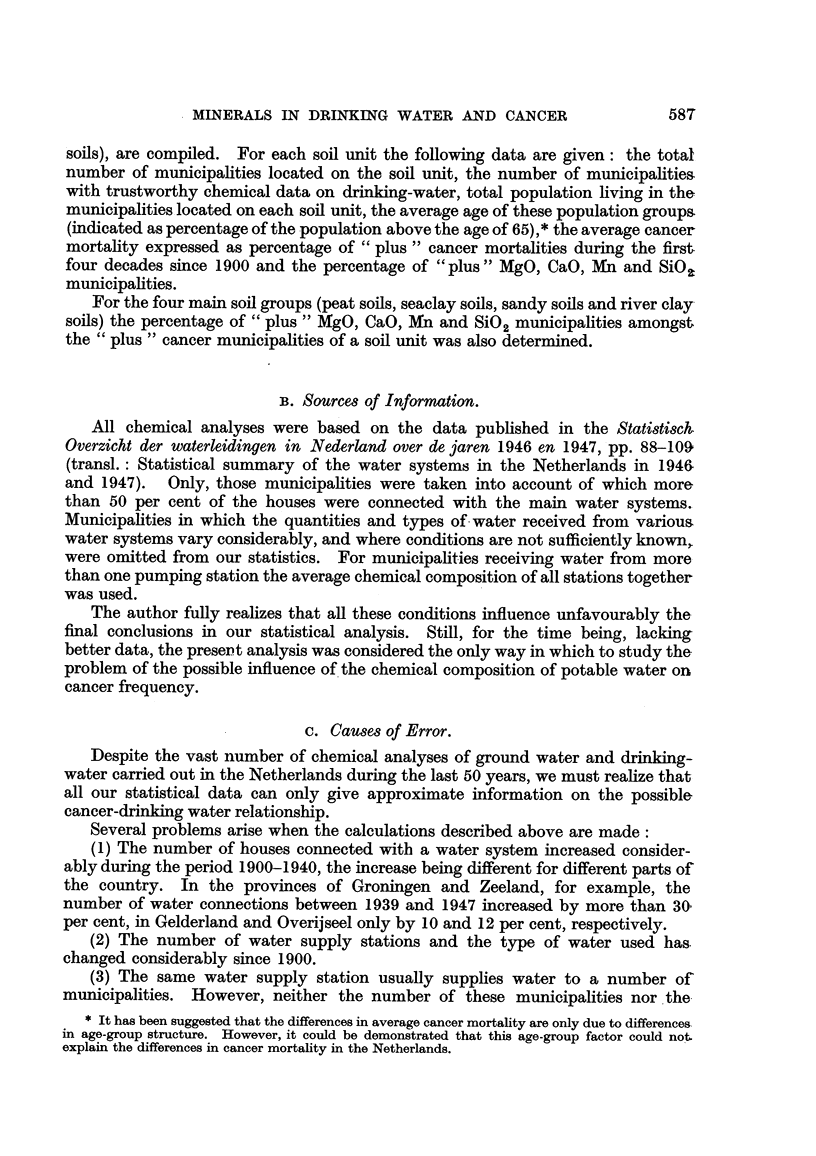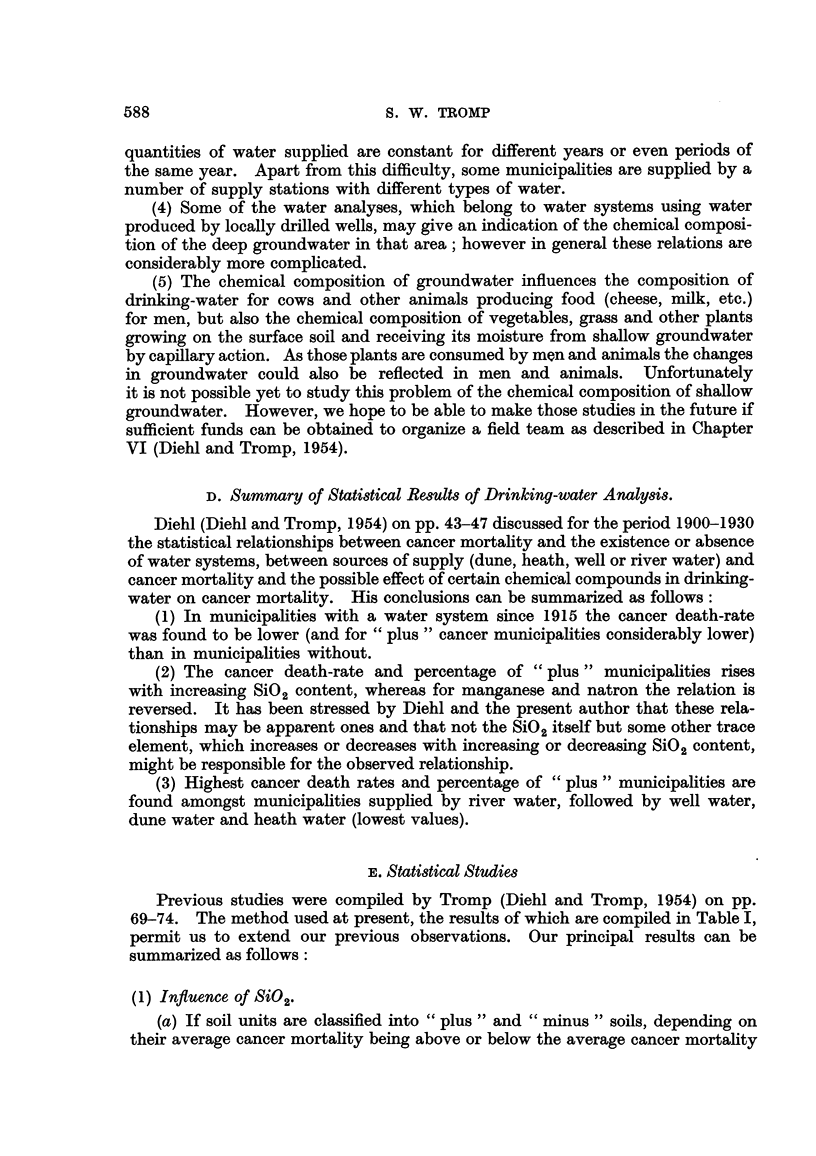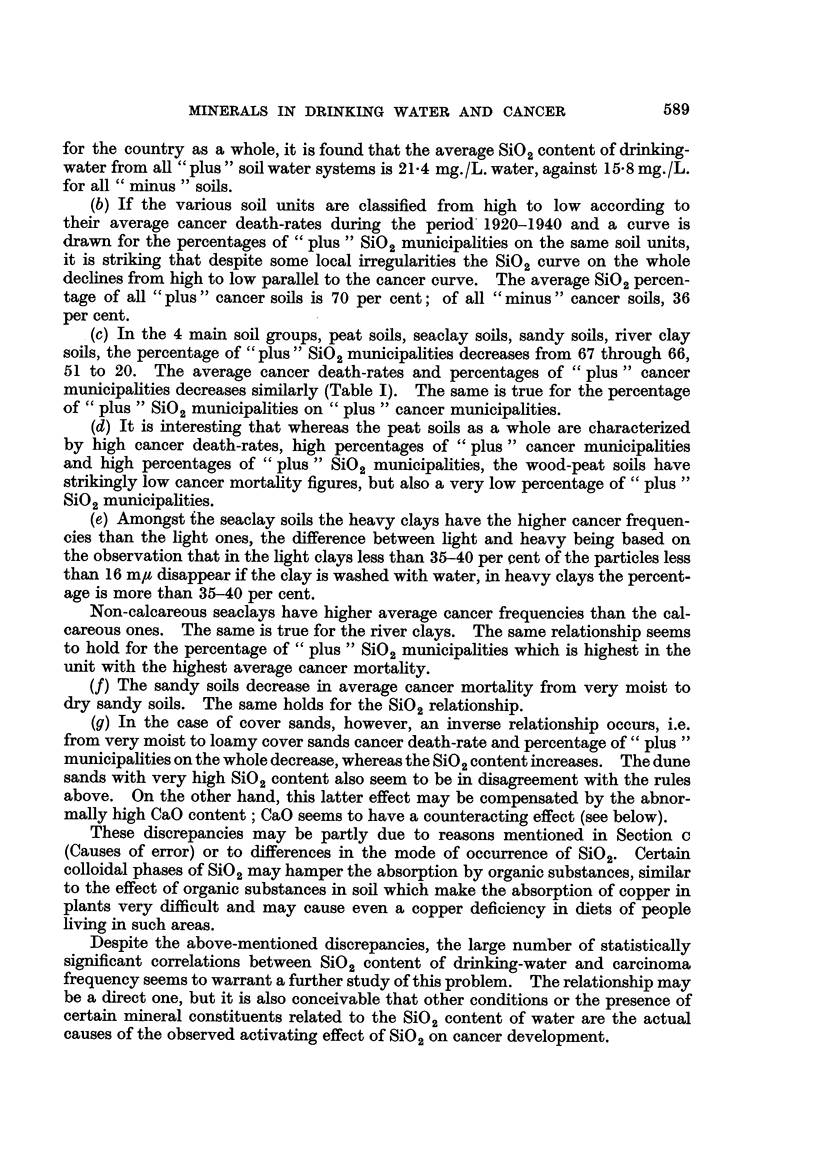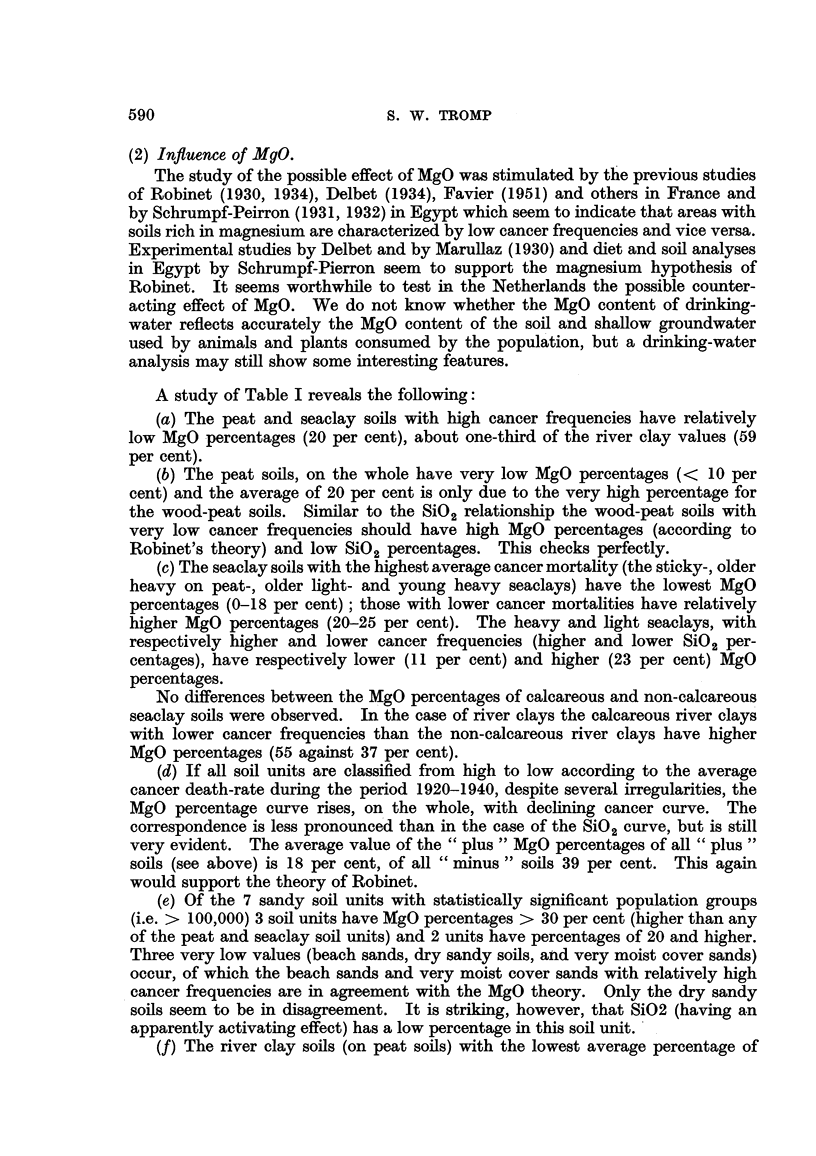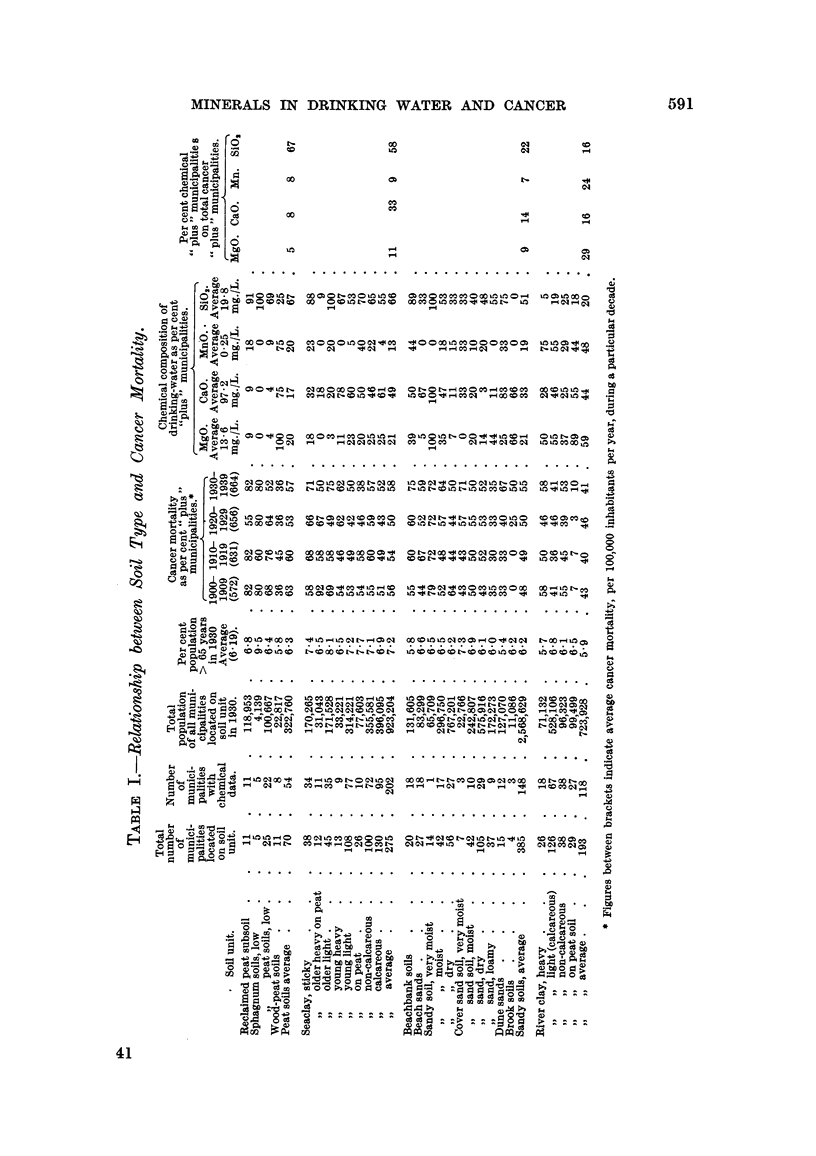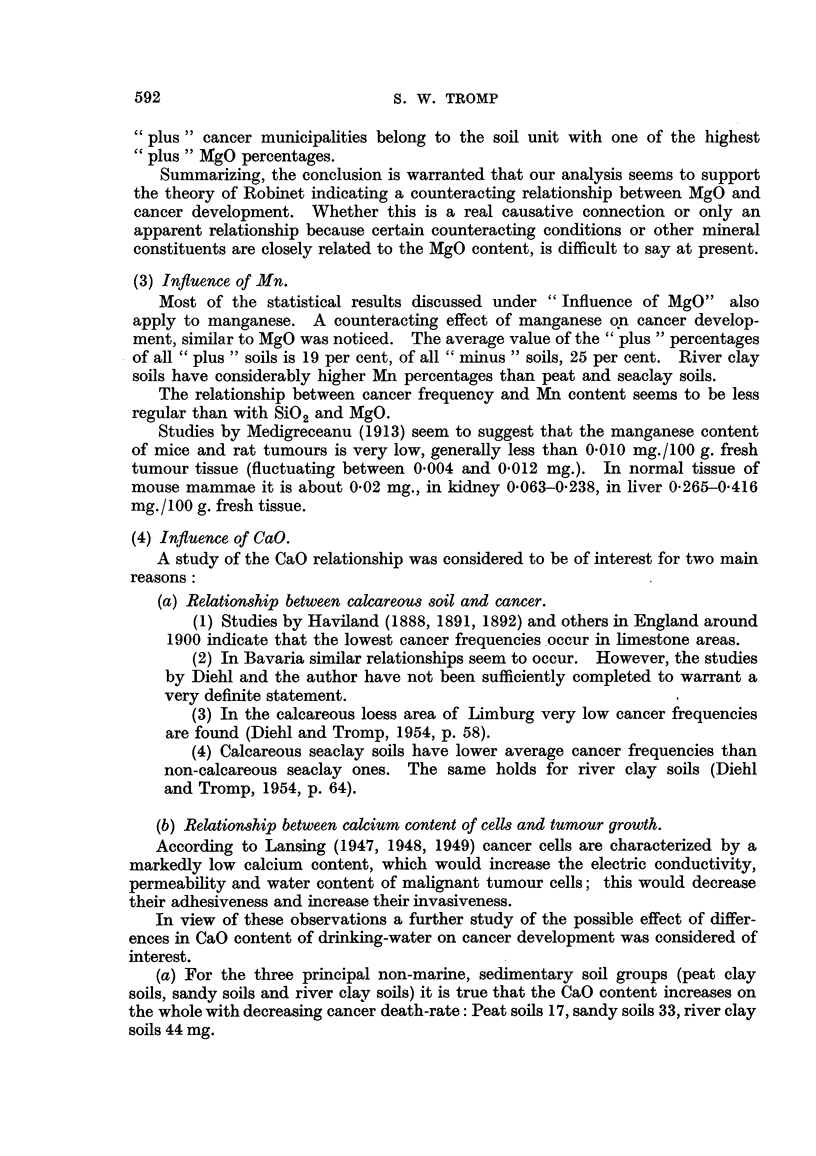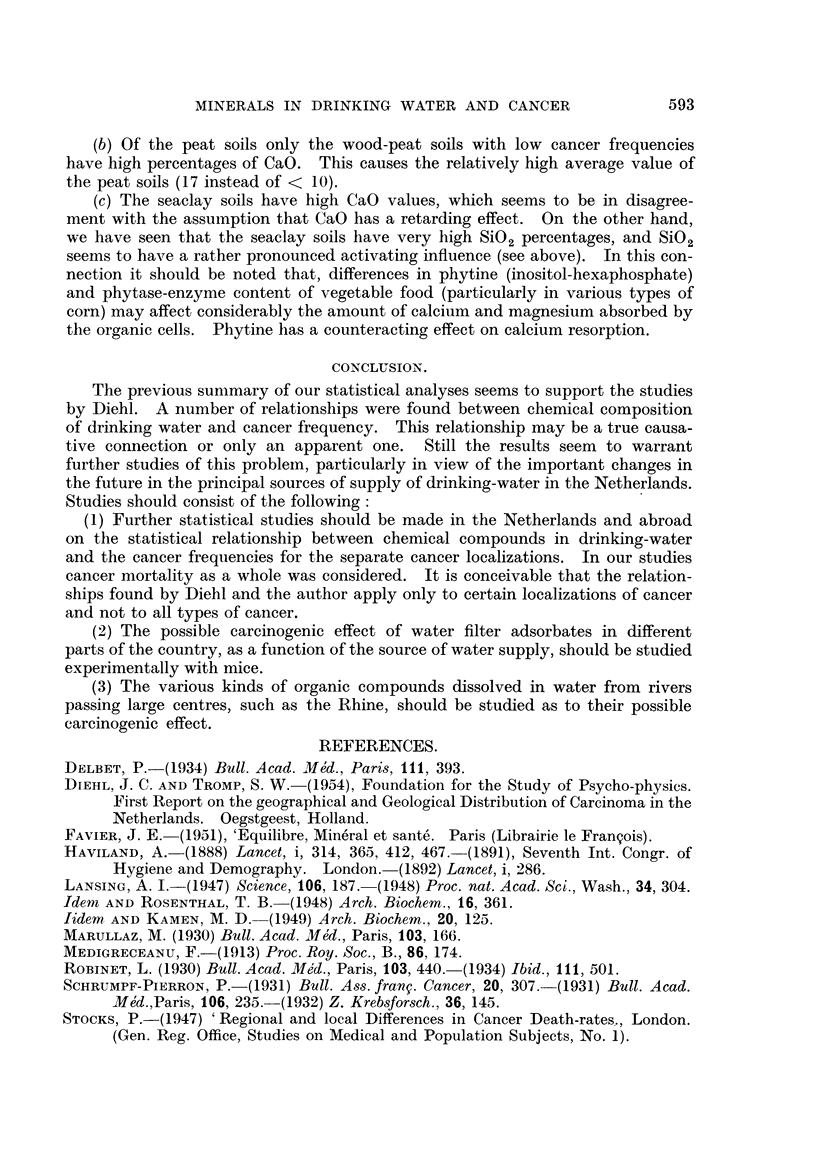# Statistical Study of the Possible Relationship between Mineral Constituents in Drinking-water and Cancer Mortality in the Netherlands (Period 1900-1940)

**DOI:** 10.1038/bjc.1954.63

**Published:** 1954-12

**Authors:** S. W. Tromp


### STATISTICAL STUDY OF THE POSSIBLE RELATIONSHIP BETWEEN MINERAL CONSTITUENTS IN DRINKING-WATER AND CANCER MORTALITY IN THE NETHERLANDS (PERIOD 1900–1940)

S. W. TROMP.

*From Hofbrouckerlaan* 54, *Oegstgeest* (*Leiden*), *Netherlands.*

Received for publication August 5, 1954.

IN view of the great shortage of drinking-water in the Netherlands, as a result of the increased consumption per inhabitant, large schemes have been projected for the future to introduce river water as the primary source of water supply. Possible biochemical effects of certain mineral constituents present in drinking-water could be considerable if a special type of water were consumed for a number of years.

Whereas the biological effect of iodine, and a number of other chemical elements, on the health condition of the human body is generally agreed upon, the possible effect of drinking-water on the development of cancer is unknown and therefore the effect of the new water projects on the health of a great part of the nation seems to be uncertain.

Recent experiments with mice in the U.S.A. suggest that there are carcinogenic properties of certain carbon adsorbates in various sources of water supply, depending upon the type of industrial wastes released into the water.

Studies by Stocks (1947), formerly Chief Medical Statistician of the General Register Office in London, indicate a peculiar distribution of cancer in Greater London which could not be explained by differences in age groups, social conditions, number of hours of sunshine, etc. Stocks (1947) noticed that the four London boroughs supplied largely by well-water had lower cancer mortalities than most of the boroughs supplied by river water. Although the observed correlation does not prove the existence of a real causative connection, it certainly supports the necessity of a closer study of the water-cancer problem.

### *Statistical Water-cancer Analysis in the Netherlands.*

The reasons given above prompted Dr. J. C. Diehl (formerly Surgeon-General of the Netherlands Army) and the author to study for the Netherlands the possible relationship between the physico-chemical properties of drinking-water and cancer mortality. The studies were carried out independently since the authors became acquainted only in 1952. Yet their general conclusions were in agreement. These drinking-water studies were part of a more extensive study by Diehl (since 1935) and the author (since 1946) on the geographical and geological distribution of cancer in the Netherlands, the results of which have been recently published (Diehl and Tromp, 1954).

### A. *Methods Applied.*

In the previous studies by the author all municipalities in the Netherlands (about 1,000) were classified according to the various soil units on which the principal part of the municipalities was located. The principal soil type for each municipality was determined through the kind intermediary of the Agricultural Soil Survey Institute at Wageningen. Altogether 62 soil units were used in our classification which could be grouped into larger units. For the municipalities located on the same soil unit the average amount (in milligrams per litre) of certain chemical compounds present in the drinking-water consumed by those municipalities was calculated. For these initial studies only the compounds CaO, SiO2, MgO and Mn were used. The result of these analyses are published on p. 72 and in Annex No. 2 of the publication by Diehl and Tromp (1954).

The average chemical composition of drinking-water for a particular soil unit could be influenced considerably by a few extreme figures, either very high or very low quantities of a certain compound present in the drinking-water of one or two municipalities only. This complicates considerably the cancer-water analysis. In view of this difficulty not the average composition but the number and percentage of " plus " municipalities was determined in the present analysis, i.e. for each soil unit the percentage of municipalities connected with a water system (located on that particular soil unit), with a chemical composition of drinking-water above the average for the country, was calculated in respect to the total number of municipalities connected with a water system located on that soil unit. It should be noted that, as indicated in Table I, the total number of municipalities located on a soil unit is usually larger than the number of municipalities connected with a water system. For the purpose of this investigation the average amount of MgO, CaO, Mn and $SiO_2$ in the drinking-water of all municipalities with water systems was first determined by adding the amounts of MgO (CaO, MnO and $SiO_2$ respectively) in milligrams per litre water in the various municipalities and by dividing this total amount by the number of municipalities. The fact that in some of the " plus " municipalities the MgO content is very high above the average explains the peculiar distribution on each side of the " average " as in the case of MgO (29 per cent " plus " against 71 per cent " minus " on a total of 522 municipalities).

The next step was to determine for each soil unit the number of municipalities with drinking-water containing those four compounds in amounts exceeding the average for the country as a whole (this was done for each compound separately) and to recalculate these numbers into percentages.

For our comparison between drinking-water analysis and average cancer mortality in municipalities located on the various soil units the average yearly cancer death-rate per 100,000 inhabitants was purposely not used, but the percentage of "plus" cancer municipalities (i.e. municipalities with cancer death rates above the average for the country) per soil unit was determined. For soil units with large population groups (over 100,000) the average cancer death-rate runs parallel to the percentage of " plus " municipalities. However, in small population groups considerable deviations may occur because the statistical results are not trustworthy.

In Table I, 27 principal soil units with large population groups, which can be united into 4 very large groups (peat soils, seaclay soils, sandy soils and river clay soils), are compiled. For each soil unit the following data are given: the total number of municipalities located on the soil unit, the number of municipalities with trustworthy chemical data on drinking-water, total population living in the municipalities located on each soil unit, the average age of these population groups (indicated as percentage of the population above the age of 65),* the average cancer mortality expressed as percentage of "plus" cancer mortalities during the first four decades since 1900 and the percentage of "plus" MgO, CaO, Mn and $SiO_2$ municipalities.

For the four main soil groups (peat soils, seaclay soils, sandy soils and river clay soils) the percentage of "plus" MgO, CaO, Mn and $SiO_2$ municipalities amongst the "plus" cancer municipalities of a soil unit was also determined.

### B. *Sources of Information.*

All chemical analyses were based on the data published in the *Statistisch Overzicht der waterleidingen in Nederland over de jaren* 1946 *en* 1947, pp. 88–109 (transl.: Statistical summary of the water systems in the Netherlands in 1946 and 1947). Only, those municipalities were taken into account of which more than 50 per cent of the houses were connected with the main water systems. Municipalities in which the quantities and types of water received from various water systems vary considerably, and where conditions are not sufficiently known, were omitted from our statistics. For municipalities receiving water from more than one pumping station the average chemical composition of all stations together was used.

The author fully realizes that all these conditions influence unfavourably the final conclusions in our statistical analysis. Still, for the time being, lacking better data, the present analysis was considered the only way in which to study the problem of the possible influence of the chemical composition of potable water on cancer frequency.

### C. *Causes of Error.*

Despite the vast number of chemical analyses of ground water and drinking-water carried out in the Netherlands during the last 50 years, we must realize that all our statistical data can only give approximate information on the possible cancer-drinking water relationship.

Several problems arise when the calculations described above are made:

(1) The number of houses connected with a water system increased considerably during the period 1900–1940, the increase being different for different parts of the country. In the provinces of Groningen and Zeeland, for example, the number of water connections between 1939 and 1947 increased by more than 30 per cent, in Gelderland and Overijseel only by 10 and 12 per cent, respectively.

(2) The number of water supply stations and the type of water used has changed considerably since 1900.

(3) The same water supply station usually supplies water to a number of municipalities. However, neither the number of these municipalities nor the

* It has been suggested that the differences in average cancer mortality are only due to differences in age-group structure. However, it could be demonstrated that this age-group factor could not explain the differences in cancer mortality in the Netherlands.

quantities of water supplied are constant for different years or even periods of the same year. Apart from this difficulty, some municipalities are supplied by a number of supply stations with different types of water.

(4) Some of the water analyses, which belong to water systems using water produced by locally drilled wells, may give an indication of the chemical composition of the deep groundwater in that area ; however in general these relations are considerably more complicated.

(5) The chemical composition of groundwater influences the composition of drinking-water for cows and other animals producing food (cheese, milk, etc.) for men, but also the chemical composition of vegetables, grass and other plants growing on the surface soil and receiving its moisture from shallow groundwater by capillary action. As those plants are consumed by men and animals the changes in groundwater could also be reflected in men and animals. Unfortunately it is not possible yet to study this problem of the chemical composition of shallow groundwater. However, we hope to be able to make those studies in the future if sufficient funds can be obtained to organize a field team as described in Chapter VI (Diehl and Tromp, 1954).

### D. *Summary of Statistical Results of Drinking-water Analysis.*

Diehl (Diehl and Tromp, 1954) on pp. 43–47 discussed for the period 1900–1930 the statistical relationships between cancer mortality and the existence or absence of water systems, between sources of supply (dune, heath, well or river water) and cancer mortality and the possible effect of certain chemical compounds in drinking-water on cancer mortality. His conclusions can be summarized as follows :

(1) In municipalities with a water system since 1915 the cancer death-rate was found to be lower (and for " plus " cancer municipalities considerably lower) than in municipalities without.

(2) The cancer death-rate and percentage of " plus " municipalities rises with increasing $SiO_2$ content, whereas for manganese and natron the relation is reversed. It has been stressed by Diehl and the present author that these relationships may be apparent ones and that not the $SiO_2$ itself but some other trace element, which increases or decreases with increasing or decreasing $SiO_2$ content, might be responsible for the observed relationship.

(3) Highest cancer death rates and percentage of " plus " municipalities are found amongst municipalities supplied by river water, followed by well water, dune water and heath water (lowest values).

### E. *Statistical Studies*

Previous studies were compiled by Tromp (Diehl and Tromp, 1954) on pp. 69–74. The method used at present, the results of which are compiled in Table I, permit us to extend our previous observations. Our principal results can be summarized as follows :

(1) *Influence of $SiO_2$.*

(*a*) If soil units are classified into " plus " and " minus " soils, depending on their average cancer mortality being above or below the average cancer mortality for the country as a whole, it is found that the average $SiO_2$ content of drinking-water from all "plus" soil water systems is 21·4 mg./L. water, against 15·8 mg./L. for all "minus" soils.

(*b*) If the various soil units are classified from high to low according to their average cancer death-rates during the period 1920–1940 and a curve is drawn for the percentages of "plus" $SiO_2$ municipalities on the same soil units, it is striking that despite some local irregularities the $SiO_2$ curve on the whole declines from high to low parallel to the cancer curve. The average $SiO_2$ percentage of all "plus" cancer soils is 70 per cent; of all "minus" cancer soils, 36 per cent.

(*c*) In the 4 main soil groups, peat soils, seaclay soils, sandy soils, river clay soils, the percentage of "plus" $SiO_2$ municipalities decreases from 67 through 66, 51 to 20. The average cancer death-rates and percentages of "plus" cancer municipalities decreases similarly (Table I). The same is true for the percentage of "plus" $SiO_2$ municipalities on "plus" cancer municipalities.

(*d*) It is interesting that whereas the peat soils as a whole are characterized by high cancer death-rates, high percentages of "plus" cancer municipalities and high percentages of "plus" $SiO_2$ municipalities, the wood-peat soils have strikingly low cancer mortality figures, but also a very low percentage of "plus" $SiO_2$ municipalities.

(*e*) Amongst the seaclay soils the heavy clays have the higher cancer frequencies than the light ones, the difference between light and heavy being based on the observation that in the light clays less than 35–40 per cent of the particles less than 16 m$\mu$ disappear if the clay is washed with water, in heavy clays the percentage is more than 35–40 per cent.

Non-calcareous seaclays have higher average cancer frequencies than the calcareous ones. The same is true for the river clays. The same relationship seems to hold for the percentage of "plus" $SiO_2$ municipalities which is highest in the unit with the highest average cancer mortality.

(*f*) The sandy soils decrease in average cancer mortality from very moist to dry sandy soils. The same holds for the $SiO_2$ relationship.

(*g*) In the case of cover sands, however, an inverse relationship occurs, i.e. from very moist to loamy cover sands cancer death-rate and percentage of "plus" municipalities on the whole decrease, whereas the $SiO_2$ content increases. The dune sands with very high $SiO_2$ content also seem to be in disagreement with the rules above. On the other hand, this latter effect may be compensated by the abnormally high CaO content ; CaO seems to have a counteracting effect (see below).

These discrepancies may be partly due to reasons mentioned in Section C (Causes of error) or to differences in the mode of occurrence of $SiO_2$. Certain colloidal phases of $SiO_2$ may hamper the absorption by organic substances, similar to the effect of organic substances in soil which make the absorption of copper in plants very difficult and may cause even a copper deficiency in diets of people living in such areas.

Despite the above-mentioned discrepancies, the large number of statistically significant correlations between $SiO_2$ content of drinking-water and carcinoma frequency seems to warrant a further study of this problem. The relationship may be a direct one, but it is also conceivable that other conditions or the presence of certain mineral constituents related to the $SiO_2$ content of water are the actual causes of the observed activating effect of $SiO_2$ on cancer development.

(2) *Influence of MgO.*

The study of the possible effect of MgO was stimulated by the previous studies of Robinet (1930, 1934), Delbet (1934), Favier (1951) and others in France and by Schrumpf-Peirron (1931, 1932) in Egypt which seem to indicate that areas with soils rich in magnesium are characterized by low cancer frequencies and vice versa. Experimental studies by Delbet and by Marullaz (1930) and diet and soil analyses in Egypt by Schrumpf-Pierron seem to support the magnesium hypothesis of Robinet. It seems worthwhile to test in the Netherlands the possible counteracting effect of MgO. We do not know whether the MgO content of drinking-water reflects accurately the MgO content of the soil and shallow groundwater used by animals and plants consumed by the population, but a drinking-water analysis may still show some interesting features.

A study of Table I reveals the following:

(*a*) The peat and seaclay soils with high cancer frequencies have relatively low MgO percentages (20 per cent), about one-third of the river clay values (59 per cent).

(*b*) The peat soils, on the whole have very low MgO percentages ($<$ 10 per cent) and the average of 20 per cent is only due to the very high percentage for the wood-peat soils. Similar to the $SiO_2$ relationship the wood-peat soils with very low cancer frequencies should have high MgO percentages (according to Robinet's theory) and low $SiO_2$ percentages. This checks perfectly.

(*c*) The seaclay soils with the highest average cancer mortality (the sticky-, older heavy on peat-, older light- and young heavy seaclays) have the lowest MgO percentages (0–18 per cent); those with lower cancer mortalities have relatively higher MgO percentages (20–25 per cent). The heavy and light seaclays, with respectively higher and lower cancer frequencies (higher and lower $SiO_2$ percentages), have respectively lower (11 per cent) and higher (23 per cent) MgO percentages.

No differences between the MgO percentages of calcareous and non-calcareous seaclay soils were observed. In the case of river clays the calcareous river clays with lower cancer frequencies than the non-calcareous river clays have higher MgO percentages (55 against 37 per cent).

(*d*) If all soil units are classified from high to low according to the average cancer death-rate during the period 1920–1940, despite several irregularities, the MgO percentage curve rises, on the whole, with declining cancer curve. The correspondence is less pronounced than in the case of the $SiO_2$ curve, but is still very evident. The average value of the " plus " MgO percentages of all " plus " soils (see above) is 18 per cent, of all " minus " soils 39 per cent. This again would support the theory of Robinet.

(*e*) Of the 7 sandy soil units with statistically significant population groups (i.e. $>$ 100,000) 3 soil units have MgO percentages $>$ 30 per cent (higher than any of the peat and seaclay soil units) and 2 units have percentages of 20 and higher. Three very low values (beach sands, dry sandy soils, and very moist cover sands) occur, of which the beach sands and very moist cover sands with relatively high cancer frequencies are in agreement with the MgO theory. Only the dry sandy soils seem to be in disagreement. It is striking, however, that SiO2 (having an apparently activating effect) has a low percentage in this soil unit.

(*f*) The river clay soils (on peat soils) with the lowest average percentage of

TABLE I.—*Relationship between Soil Type and Cancer Mortality.*

| Soil unit. | Total number of municipalities located on soil unit. | Number of municipalities with chemical data. | Total population of all municipalities located on soil unit in 1930. | Per cent population > 65 years in 1930 Average (6·19). | Cancer mortality as per cent "plus" municipalities.* 1900–1909 (572) | 1910–1919 (631) | 1920–1929 (656) | 1930–1939 (664) | Chemical composition of drinking-water as per cent "plus" municipalities. MgO. Average 13·6 mg./L. | CaO. Average 97·2 mg./L. | MnO. Average 0·25 mg./L. | $SiO_2$. Average 19·8 mg./L. | Per cent chemical "plus" municipalities on total cancer "plus" municipalities. MgO. | CaO. | Mn. | $SiO_2$ |
|---|---|---|---|---|---|---|---|---|---|---|---|---|---|---|---|---|
| Reclaimed peat subsoil | 11 | 11 | 118,953 | 6·8 | 82 | 82 | 55 | 82 | 9 | 9 | 18 | 91 | | | | |
| Sphagnum soils, low | 5 | 5 | 4,139 | 9·5 | 80 | 60 | 80 | 80 | 0 | 0 | 0 | 100 | | | | |
| " peat soils, low | 25 | 22 | 100,667 | 6·4 | 68 | 76 | 64 | 52 | 4 | 4 | 9 | 69 | | | | |
| Wood-peat soils | 11 | 8 | 22,817 | 5·8 | 36 | 45 | 36 | 36 | 100 | 75 | 75 | 25 | | | | |
| Peat soils average | 70 | 54 | 322,760 | 6·3 | 63 | 60 | 53 | 57 | 20 | 17 | 20 | 67 | 5 | 8 | 8 | 67 |
| Seaclay, sticky | 38 | 34 | 170,265 | 7·4 | 58 | 68 | 66 | 71 | 18 | 32 | 23 | 88 | | | | |
| " older heavy on peat | 12 | 11 | 31,043 | 6·5 | 92 | 58 | 67 | 50 | 0 | 18 | 0 | 9 | | | | |
| " older light | 45 | 35 | 171,528 | 8·1 | 69 | 58 | 49 | 75 | 3 | 20 | 20 | 100 | | | | |
| " young heavy | 13 | 9 | 33,221 | 6·5 | 54 | 46 | 62 | 62 | 11 | 78 | 0 | 67 | | | | |
| " young light | 108 | 77 | 314,221 | 7·2 | 53 | 49 | 42 | 50 | 23 | 60 | 5 | 53 | | | | |
| " on peat | 26 | 10 | 77,603 | 7·7 | 54 | 58 | 46 | 38 | 20 | 50 | 40 | 70 | | | | |
| " non-calcareous | 100 | 72 | 355,581 | 7·1 | 55 | 60 | 59 | 57 | 25 | 46 | 22 | 65 | | | | |
| " calcareous | 130 | 95 | 396,095 | 6·9 | 51 | 49 | 43 | 52 | 25 | 61 | 4 | 55 | | | | |
| " average | 275 | 202 | 923,204 | 7·2 | 56 | 54 | 50 | 58 | 21 | 49 | 13 | 66 | 11 | 33 | 9 | 58 |
| Beachbank soils | 20 | 18 | 131,605 | 5·8 | 55 | 60 | 60 | 75 | 39 | 50 | 44 | 89 | | | | |
| Beach sands | 27 | 18 | 83,299 | 6·6 | 44 | 67 | 52 | 59 | 5 | 67 | 0 | 33 | | | | |
| Sandy soil, very moist | 14 | 1 | 65,709 | 6·5 | 79 | 72 | 72 | 72 | 100 | 100 | 0 | 100 | | | | |
| " " moist | 42 | 17 | 296,750 | 6·5 | 52 | 48 | 57 | 64 | 35 | 47 | 18 | 53 | | | | |
| " " dry | 56 | 27 | 767,201 | 6·2 | 64 | 44 | 44 | 50 | 7 | 11 | 15 | 33 | | | | |
| Cover sand soil, very moist | 7 | 3 | 22,766 | 7·3 | 43 | 43 | 57 | 71 | 0 | 33 | 33 | 33 | | | | |
| " sand soil, moist | 42 | 10 | 242,807 | 6·9 | 50 | 50 | 55 | 50 | 20 | 20 | 10 | 40 | | | | |
| " sand, dry | 105 | 29 | 575,916 | 6·1 | 43 | 52 | 53 | 52 | 14 | 3 | 20 | 48 | | | | |
| " sand, loamy | 37 | 9 | 172,273 | 6·0 | 35 | 30 | 33 | 35 | 44 | 11 | 0 | 55 | | | | |
| Dune sands | 15 | 12 | 127,070 | 5·4 | 33 | 33 | 40 | 67 | 25 | 83 | 33 | 75 | | | | |
| Brook soils | 4 | 3 | 11,086 | 6·2 | 0 | 0 | 25 | 50 | 66 | 66 | 0 | 0 | | | | |
| Sandy soils, average | 385 | 148 | 2,568,629 | 6·2 | 48 | 49 | 50 | 55 | 21 | 33 | 19 | 51 | 9 | 14 | 7 | 22 |
| River clay, heavy | 26 | 18 | 71,132 | 5·7 | 58 | 50 | 46 | 58 | 50 | 28 | 75 | 5 | | | | |
| " " light (calcareous) | 126 | 67 | 528,106 | 6·8 | 41 | 36 | 46 | 41 | 55 | 46 | 55 | 19 | | | | |
| " " non-calcareous | 38 | 38 | 96,323 | 6·1 | 55 | 45 | 39 | 53 | 37 | 25 | 29 | 25 | | | | |
| " " on peat soil | 29 | 27 | 99,499 | 6·5 | 7 | 7 | 3 | 10 | 89 | 55 | 44 | 18 | | | | |
| " " average | 193 | 118 | 723,928 | 5·9 | 43 | 40 | 46 | 41 | 59 | 44 | 48 | 20 | 29 | 16 | 24 | 16 |

* Figures between brackets indicate average cancer mortality, per 100,000 inhabitants per year, during a particular decade.

" plus " cancer municipalities belong to the soil unit with one of the highest " plus " MgO percentages.

Summarizing, the conclusion is warranted that our analysis seems to support the theory of Robinet indicating a counteracting relationship between MgO and cancer development. Whether this is a real causative connection or only an apparent relationship because certain counteracting conditions or other mineral constituents are closely related to the MgO content, is difficult to say at present.

(3) *Influence of Mn.*

Most of the statistical results discussed under " Influence of MgO" also apply to manganese. A counteracting effect of manganese on cancer development, similar to MgO was noticed. The average value of the " plus " percentages of all " plus " soils is 19 per cent, of all " minus " soils, 25 per cent. River clay soils have considerably higher Mn percentages than peat and seaclay soils.

The relationship between cancer frequency and Mn content seems to be less regular than with $SiO_2$ and MgO.

Studies by Medigreceanu (1913) seem to suggest that the manganese content of mice and rat tumours is very low, generally less than 0·010 mg./100 g. fresh tumour tissue (fluctuating between 0·004 and 0·012 mg.). In normal tissue of mouse mammae it is about 0·02 mg., in kidney 0·063–0·238, in liver 0·265–0·416 mg./100 g. fresh tissue.

(4) *Influence of CaO.*

A study of the CaO relationship was considered to be of interest for two main reasons :

(*a*) *Relationship between calcareous soil and cancer.*

(1) Studies by Haviland (1888, 1891, 1892) and others in England around 1900 indicate that the lowest cancer frequencies occur in limestone areas.

(2) In Bavaria similar relationships seem to occur. However, the studies by Diehl and the author have not been sufficiently completed to warrant a very definite statement.

(3) In the calcareous loess area of Limburg very low cancer frequencies are found (Diehl and Tromp, 1954, p. 58).

(4) Calcareous seaclay soils have lower average cancer frequencies than non-calcareous seaclay ones. The same holds for river clay soils (Diehl and Tromp, 1954, p. 64).

(*b*) *Relationship between calcium content of cells and tumour growth.*

According to Lansing (1947, 1948, 1949) cancer cells are characterized by a markedly low calcium content, which would increase the electric conductivity, permeability and water content of malignant tumour cells; this would decrease their adhesiveness and increase their invasiveness.

In view of these observations a further study of the possible effect of differences in CaO content of drinking-water on cancer development was considered of interest.

(*a*) For the three principal non-marine, sedimentary soil groups (peat clay soils, sandy soils and river clay soils) it is true that the CaO content increases on the whole with decreasing cancer death-rate: Peat soils 17, sandy soils 33, river clay soils 44 mg.

(*b*) Of the peat soils only the wood-peat soils with low cancer frequencies have high percentages of CaO. This causes the relatively high average value of the peat soils (17 instead of $< 10$).

(*c*) The seaclay soils have high CaO values, which seems to be in disagreement with the assumption that CaO has a retarding effect. On the other hand, we have seen that the seaclay soils have very high $SiO_2$ percentages, and $SiO_2$ seems to have a rather pronounced activating influence (see above). In this connection it should be noted that, differences in phytine (inositol-hexaphosphate) and phytase-enzyme content of vegetable food (particularly in various types of corn) may affect considerably the amount of calcium and magnesium absorbed by the organic cells. Phytine has a counteracting effect on calcium resorption.

### CONCLUSION.

The previous summary of our statistical analyses seems to support the studies by Diehl. A number of relationships were found between chemical composition of drinking water and cancer frequency. This relationship may be a true causative connection or only an apparent one. Still the results seem to warrant further studies of this problem, particularly in view of the important changes in the future in the principal sources of supply of drinking-water in the Netherlands. Studies should consist of the following :

(1) Further statistical studies should be made in the Netherlands and abroad on the statistical relationship between chemical compounds in drinking-water and the cancer frequencies for the separate cancer localizations. In our studies cancer mortality as a whole was considered. It is conceivable that the relationships found by Diehl and the author apply only to certain localizations of cancer and not to all types of cancer.

(2) The possible carcinogenic effect of water filter adsorbates in different parts of the country, as a function of the source of water supply, should be studied experimentally with mice.

(3) The various kinds of organic compounds dissolved in water from rivers passing large centres, such as the Rhine, should be studied as to their possible carcinogenic effect.

### REFERENCES.

Delbet, P.—(1934) *Bull. Acad. Méd., Paris*, **111**, 393.

Diehl, J. C. and Tromp, S. W.—(1954), Foundation for the Study of Psycho-physics. First Report on the geographical and Geological Distribution of Carcinoma in the Netherlands. Oegstgeest, Holland.

Favier, J. E.—(1951), 'Equilibre, Minéral et santé. Paris (Librairie le François).

Haviland, A.—(1888) *Lancet*, i, 314, 365, 412, 467.—(1891), Seventh Int. Congr. of Hygiene and Demography. London.—(1892) *Lancet*, i, 286.

Lansing, A. I.—(1947) *Science*, **106**, 187.—(1948) *Proc. nat. Acad. Sci.*, Wash., **34**, 304.

*Idem* and Rosenthal, T. B.—(1948) *Arch. Biochem.*, **16**, 361.

*Iidem* and Kamen, M. D.—(1949) *Arch. Biochem.*, **20**, 125.

Marullaz, M. (1930) *Bull. Acad. Méd.*, Paris, **103**, 166.

Medigreceanu, F.—(1913) *Proc. Roy. Soc.*, B., **86**, 174.

Robinet, L. (1930) *Bull. Acad. Méd.*, Paris, **103**, 440.—(1934) *Ibid.*, **111**, 501.

Schrumpf-Pierron, P.—(1931) *Bull. Ass. franç. Cancer*, **20**, 307.—(1931) *Bull. Acad. Méd.*,Paris, **106**, 235.—(1932) *Z. Krebsforsch.*, **36**, 145.

Stocks, P.—(1947) 'Regional and local Differences in Cancer Death-rates., London. (Gen. Reg. Office, Studies on Medical and Population Subjects, No. 1).